# Bedside teaching: an underutilized tool in medical education

**DOI:** 10.5116/ijme.5780.bdba

**Published:** 2016-08-07

**Authors:** Mohammed Garout, Abdulelah Nuqali, Ahmad Alhazmi, Hani Almoallim

**Affiliations:** 1 Department of Community Medicine and Pilgrims Healthcare, Umm Al-Qura University, Makkah, Saudi Arabia; 2 Department of Medicine, George Washington University, Washington, DC, USA; 3 Department of Internal Medicine, Umm Al-Qura University, Makkah, Saudi Arabia; 4 Department of Surgery, Umm Al-Qura University, Makkah, Saudi Arabia

**Keywords:** Bedside teaching, tool in medical education, learning triad, BST, patients, Saudi Arabia, USA

## Introduction

Bedside teaching (BST) is a fundamental component of clinical training and an essential tool in the creation of a competent physician.^1^^-^^15^ Sir William Osler (1849-1919), one of Canada’s most renowned physicians, was the first to introduce BST to medical education in 1892. He described modern medical education as something that needed to be taught at the bedside: “Medicine is learned by the bedside and not in the classroom.”^9^ BST allows the physician and patient to interact at the bedside; through this physician-patient interaction process, medical students and residents are simultaneously afforded the opportunity to learn clinical skills, clinical reasoning, physician-patient communication, empathy, and professionalism.^6^^,^^12^^,^^15^

In real practice, comprehensive history taking can help the physician diagnose up to 56% of patient problems, which may rise to 73% if a physical examination is added.^8^ Much information can be gained and a proper diagnosis reached by obtaining a good medical history and performing an efficient clinical examination.^8^ Clinical teaching in which the patient is involved is enriched by these visual, auditory, and tactile experiences.

Senior medical students and medical residents believe that BST is a valuable but underutilized tool.^15^ Time spent on BST has been on the decline since 1978, as highlighted by Ahmed, who reported that the proportion of teaching time taken up by BST had declined from 75% 30 years ago to only 16% today.^8^

### The learning triad

The BST learning triad comprises patients, students, and tutors.^6^ All three must be present for BST to occur and it must occur within a clinical environment. Each individual member brings his or her own value to the learning triad. For example, the student brings medical knowledge and the eagerness to learn; the tutor brings depth of knowledge, mentorship, and willingness to help the student learn and make connections; and finally, the patient brings relevant clinical issues to the forefront that allow the student to learn. An effective learning environment requires all three groups to work together in the learning triad.^6^ The obstacles that may reduce the effectiveness of BST can be categorized by each group in the learning triad.

### Patients

Patients should be actively involved in BST and are the cornerstones of this type of learning. Educators and medical students assume that BST may put patients under stress and embarrass them.^9^^,^^16^ Nair and colleagues found, however, that 77% of patients enjoy BST and 83% stated that it did not make them anxious.^9^ It is recommended that patients be asked for permission before teaching starts and prior to any physical examination, which they have a right to refuse at any time.^15^ Ensuring that BST occurs at the patient’s discretion is important, especially in multi-rooms, by using curtains or barriers to maximize privacy.^17^ Moreover, it is essential to consider the patient’s health status, especially if the individual is very ill, and to respect his or her choice if the patient wants to discontinue the session.

Obstacles that are described in the literature regarding patient behavior that could influence BST include lack of cooperation,^5^ fear of embracement,^9^^,^^15^^,^^16^ and misinterpretation of discussion and cultural issues.^10^ Overcoming these obstacles requires the patient’s role being changed from simply an interesting case into that of an active participant who has the full right to discuss, interrupt, and offer deep and broad insights into his or her illness.^6^^,^^10^^,^^12^ Patients must be informed, have the educational activity explained to them properly, and be involved in the entire process of BST; it is important to respect the learner-patient relationship and the patient’s autonomy.^12^ Finally, asking for feedback from patients at the end of BST is an essential part of involving them in the process and is highly beneficial for students when assessing their performance in such sessions.^12^

### Students

Students play an essential role in BST. They contribute to BST by bolstering its effectiveness through positive preparation prior to going on rounds and seeing patients. In addition to obtaining knowledge and being prepared before seeing patients, it is essential that students have strong communication skills to relate to patients and to collaborate with them.^5^^,^^13^ Communication skills should be reemphasized prior to entering a BST environment because it is critical that students be able to cooperate with patients in order to learn from them and assist in treating them at the bedside. Therefore, screening of communication skills is required prior to BST for any student learning, thus ensuring success for all involved.  For students to succeed, they need to set their learning goals for BST in advance and to discuss these goals with the attending physician (tutor).^13^ Allowing students the privilege of being part of the managing team is a crucial step. Many clinical skills such as performing diagnostics and therapeutic procedures can be taught during hospital rounds.

### Tutors

The tutor (attending physician), must have the appropriate clinical knowledge, maintain his/her information, and master the required clinical skills of a competent clinical educator.^14^ Furthermore, tutors who are also seen as teachers should encourage their students to be involved in discussions and allow them to be active and proactive learners.^13^ Spencer states that effective teaching depends mainly on the teacher’s communication skills, particularly in terms of questioning and giving good explanations as well as formative feedback.^12^ Despite all the preparation and planning, BST nonetheless takes place in the presence of patients in a clinical environment; thus, the tutor’s plans can deviate during the learning process. This can occur, for example, if the tutor has difficulty in engaging learners, is inexperienced with BST, lacks control over patient-student interactions, undergoes interruptions,^13^ must give apressured performance, or has to teach at multiple learner levels.

Gonzalo and colleagues proposed the following four key concepts for tutors to apply to the BST learning process in order to avoid hurdles for medical students: (1) Make BST trainee-specific: Ask trainees for their own learning goals and conduct BST on the basis of these goals. (2) Make BST disease-specific: Select the specific topic prior to BST and let both the trainer and the trainees read it thoroughly by using an updated resource. (3) Make BST patient-specific: Choose patients whose conditions have high educational value in the ward prior to BST. (4) Prepare mentally: Take steps to be mentally prepared for the many different tasks that might take place during BST.^5^ Finally, it is critical for the tutor to remember when teaching at multiple learner levels to invite higher level learners to teach the rest of the team.^17^ Another strategy is to discuss the topic from many perspectives, starting with the basic clinical presentation and progressing to the final guidelines in diagnosis and management. This approach offers an opportunity to involve all members in the discussion.^17^ Interruption is another factor that could compromise BST because trainees feel anxious when they are interrupted. Listening carefully to the patient’s history and assessing the physical examination findings with limited interruptions will improve the team’s overall communication.^13^

### Acknowledgments

The authors thank Alzaidi Chair of Research in Rheumatic Diseases at Umm Alqura University for supporting and supervising this paper. We also thank Dr. Allison Vanderbilt for her helpful comments on the manuscript.

### Conflicts of Interest

The authors declare that they have no conflict of interest. 
